# Dietary acid load as well as dietary phytochemical index, and association with multiple sclerosis: results from a case–control study

**DOI:** 10.1186/s40795-024-00897-z

**Published:** 2024-07-01

**Authors:** Alireza Hatami, Maryam Ahmadi-Khorram, Fatemeh Keykhaei, Mohtaram Hashemi, Reihane Javid, Mehrara Hashempour, Ali Jafarzadeh Esfehani, Mohsen Nematy

**Affiliations:** 1 Department of Nutrition, Faculty of Medicine, Mashhad University of Medical Sciences, Mashhad, 91779-48564 Iran; 2 Student Research Committee, Semnan University of Medical Sciences, Semnan, Iran; 3 Metabolic Syndrome Research Center, Mashhad University of Medical Sciences, Mashhad, Iran

**Keywords:** Autoimmune diseases, Multiple sclerosis, Neurodegeneration, Inflammation, Phytochemicals index, Dietary acid load, Iran, Food frequency questionnaire

## Abstract

**Introduction:**

Multiple sclerosis (MS) is a chronic inflammatory disease characterized by central nervous system (CNS) lesions. Although the etiology and pathogenesis of MS remains unclear, nutrition is among the environmental factors that may be involved in developing MS. Currently, no specific diet has been associated with MS. This study aimed to investigate the relationship between the dietary phytochemical index (DPI), dietary acid load (DAL), and the risk of developing MS.

**Methods:**

This case‒control study was conducted on 174 patients with MS and 171 healthy individuals in Mashhad, Iran. Data were collected using a 160-item semiquantitative food frequency questionnaire (FFQ). The study investigated the association between DPI, DAL, and MS, considering anthropometric measures, dietary intake, smoking habits, and sex. DPI, potential renal acid load (PRAL), and net endogenous acid production (NEAP), as indicators of DAL, were calculated based on the FFQ.

**Results:**

The study analyzed 345 participants, comprising 174 (50.4%) MS patients and 171 (49.6%) healthy individuals. The mean age of the participants was 32.45 ± 8.66 years. The DPI score was significantly lower among MS patients, while the NEAP and PRAL scores were significantly higher among MS patients compared to the healthy group. There was no relationship between NEAP (OR 1.001; 95% CI 0.959–1.044; *P* = 0.974) and PRAL (OR 1.019; 95% CI 0.979–1.061; *P* = 0.356) and MS incidence.

**Conclusions:**

The study found higher smoking and obesity rates in MS patients, with a reduced DPI score and increased DAL. Further studies are needed before recommending plant-based foods and dietary acid–base balance evaluation as therapeutic approach.

**Graphical Abstract:**

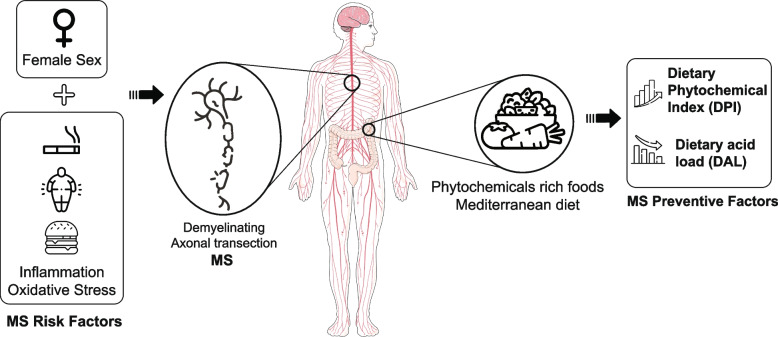

## Highlights

• This case-control study examined the relationship between DPI, dietary acid load, and chance of developing MS in 174 MS patients and 171 healthy controls.

• MS patients had significantly lower DPI scores compared to healthy controls, indicating lower intake of phytochemical-rich foods like fruits, vegetables, legumes, and whole grains.

• MS patients had significantly higher NEAP and PRAL scores compared to controls, suggesting a more acidic dietary pattern.

• Lower DPI was associated with increased chance of MS (88.2% reduction in MS for each unit increase in DPI score).

• Male sex, lower smoking rates, lower waist circumference, lower body fat percentage, higher lean mass, and lower energy intake were associated with reduced chance of MS.

• The study suggests that dietary patterns higher in phytochemical-rich plant foods and lower in acid-promoting animal proteins were related to reduced chance of MS, but further research on dietary interventions is needed.

## Introduction

Multiple sclerosis (MS) is a chronic inflammatory, neurodegenerative autoimmune disease of the central nervous system characterized by demyelination and axonal degeneration [1]. MS predominantly affects young adults, especially women aged 20–40 [2], with an increasing global incidence and prevalence [3], affecting approximately 2.8 million people worldwide in 2022 [4]. Despite global research efforts, the etiology and pathogenesis of MS remain incompletely understood [5]. However, it is well-established that both genetic and environmental factors contribute to an individual's susceptibility to developing MS [6]. Among the environmental factors, diet has garnered significant interest as a potentially modifiable risk factor that may influence the disease course and severity [7].

Previous research has explored the potential links between various dietary patterns, nutrients, and the risk of MS [8]. Plant-based dietary patterns rich in fruits, vegetables, whole grains, and legumes have been hypothesized to confer a protective effect against MS [2, 9]. This proposed benefit is attributed mainly to the abundant antioxidant and anti-inflammatory properties of phytochemicals in these plant-based food sources [10]. Conversely, dietary patterns characterized by a higher intake of animal-based protein sources have been associated with an increased risk of MS, potentially mediated through the promotion of oxidative stress, inflammation, and metabolic disturbances [11, 12].

While these previous studies have provided valuable insights, two specific dietary factors that may influence MS risk have not been extensively investigated: the dietary phytochemical index (DPI) and the dietary acid–base load (DAL). The DPI is a measure of the phytochemical content in an individual's diet, encapsulating the cumulative intake of phytochemical-rich plant foods, first proposed by McCarty [13]. On the other hand, the dietary acid load, quantified through indices such as potential renal acid load (PRAL) and net endogenous acid production (NEAP), reflects the balance between acid-producing and base-producing foods in the diet [14].

The potential mechanisms underlying the hypothesized associations between these dietary factors and MS risk are biologically plausible. Phytochemicals possess potent antioxidant and anti-inflammatory properties that may counteract the oxidative stress and chronic inflammation central to MS pathogenesis [10, 15, 16]. Conversely, a diet high in acid-promoting animal protein sources and low in alkaline plant foods may contribute to a state of low-grade metabolic acidosis, which can promote inflammation, insulin resistance, and other metabolic disturbances implicated in the development and progression of MS [11, 12, 17–20].

Despite these proposed mechanisms, the specific roles of the DPI and DAL in the context of MS risk have not been comprehensively explored. Therefore, this study aimed to investigate the relationship between the DPI, DAL (assessed by PRAL and NEAP), and the chance of developing MS in a sample of Iranian adults. By addressing this knowledge gap, the findings may provide valuable insights into potential dietary strategies that could complement traditional therapeutic approaches for this debilitating autoimmune condition.

## Methods

The study aimed to explore the association between DPI, DAL, and the chance of developing MS. It was conducted with the approval of the Ethics Committee of Mashhad University of Medical Sciences, with the reference number IR.MUMS.REC.1393.182.

### Study design and participants

This case–control study involved 197 patients with relapsing–remitting MS (RRMS), aged 18 to 65 years old. The patients were selected from the Mashhad MS Association Registry in Northeast Iran 2015. The research population included newly registered MS patients who had no change in diet for the past six months. In addition, 200 healthy individuals were included in the study. The inclusion criteria for the control group were having no neurological diseases (based on self-report), receiving services from the same hospital as the cases, and having no acute medical conditions. Exclusion criteria for the control group were pregnancy, and intentional dietary modifications. The control group was matched with the case group regarding age (in 10-year groups), sex, education level, and body mass index (BMI). The participants were given a brief explanation of how to answer the questions in the FFQ [21]. The researchers recorded the names, age, height, weight, body composition, gender, menopause, smoking habits, and educational status of the participants.

### The exclusion criteria for both groups were as follows:

[1] started to take specific diet within last 12 months, [2] taking any food supplements, and [3] under- or overestimation of energy intake (< 800 or > 4200 kcal/day). All participants provided written informed consent by completing and signing a consent form.

### Sample size

The sample size formula in correlation studies calculates the needed sample size. According to a survey conducted by Jahromi et al., the correlation coefficient for the relationship between the traditional diet score and the risk of MS was 0.27 [22].

$$n={\left[\frac{{z}_{\alpha }+{z}_{\beta }}{c}\right]}^{2}$$

$$c=0.5\times \text{ln}[\frac{1+r}{1-r}]$$

The sample size of the study included 197 patients in the case group and 200 healthy individuals in the control group, totaling 397 participants.

### Data collection tools

#### Demographic data

Skilled interviewers obtained demographic and smoking habit information.

#### Anthropometric measurements

Trained health professionals followed the CDC’s Anthropometry Procedures Manual 2007 to record anthropometric measurements for each individual as part of the National Health and Nutrition Examination Survey [23].

The participant's body composition and weight were measured while wearing minimal clothing and no shoes. A bioelectrical impedance analyzer (Tanita BC-418 Body Composition Analyzer) was used to obtain data, which was then recorded to the nearest 100 g. Their height was measured in a standing position without shoes using a tape measure with shoulders in a normal position, and the data was recorded to the nearest 0.5 cm. The waist circumference was measured twice at a level midway between the lowest rib and iliac crest using a flexible tape, and the recorded data was to the nearest millimeter. BMI was calculated using the formula: weight in kilograms divided by height in meters squared. The subjects were then classified based on their BMI using the cut-off points determined by the World Health Organization and categorized as either underweight (< 18.5 kg/m2), healthy weight (18.5–24.9 kg/m2), overweight (25–29.9 kg/m2) or obese (≥ 30 kg/m^2^) [24].

#### Assessment of dietary intake

The study assessed the participants' regular dietary intake using a semi-quantitative FFQ comprising 160 Iranian food items. The FFQ was developed and validated at Mashhad University of Medical Sciences with the correlation coefficient of 0.225 to 0.323 comparing to three-day food record for macro nutrients and 0.128 to 0.476 for micronutrients (60% overall agreement with food record) and intraclass correlation coefficient (ICC) ranging between 0.363 and 0.578 [21]. Expert dietitians conducted face-to-face personal interviews to complete the FFQ. During the interviews, household portions were confirmed through photographs to ensure accurate measurement of food intake.

The average food intake was determined based on the typical portion sizes consumed by the general Iranian population. Standard units were established using the average serving sizes of everyday food items such as a bowl of yogurt and chips, a glass of beverage, or a plate of rice. A food photo album with ten photos depicting the average portion sizes and household measures was included at the beginning of the FFQ to ensure consistency. Participants were asked about the frequency of their consumption of various food items in the past month. Their responses were categorized into four groups: never/less than once a month, monthly (1–3 times/month), weekly (1–6 times/week), and daily (1–6 times/day or more).

Portion sizes were categorized as small (less than half of the persistent moderate use), medium (equal to the determined average use), and large (one and a half times more than the moderate use or more). The frequency of consuming each food was recorded and converted to daily intake, while the serving size of the consumed items was converted to grams using household measures. The researchers neutrally asked these questions without judging the participants' eating habits.

### Dietary acid acid‒base calculation

In this study, two indices, PRAL and NEAP, were used to assess the dietary acid load according to the following formula [25, 26]:

$$\text{NEAP }(\text{mEq}/\text{d}) = [54.5 \times \text{ protein}(\text{g}/\text{d})/\text{potassium }(\text{mEq}/\text{d})] - 10.2$$

$$\text{PRAL }(\text{mEq}/\text{d}) = [\text{protein}(\text{g}/\text{d}) \times 0.49] + [\text{phosphorus}(\text{mg}/\text{d}) \times 0.037] - [\text{potassium}(\text{mg}/\text{d}) \times 0.021] - [\text{calcium }(\text{mg}/\text{d}) \times 0.013] - [\text{magnesium}(\text{mg}/\text{d}) \times 0.026]$$

The values of the required macro- and micronutrients were obtained from the FFQ.

### DPI calculation

The DPI was calculated according to the method developed by McCarty [13]. The index consists of eight components: fruits, vegetables, legumes, whole grains, soy products, nuts, seeds, olive, and olive oil. First, the energy ratio obtained from the eight foods above or food groups (kcal) to total daily energy intake was calculated and multiplied by 100. Because of the high phytochemical content of natural fruit juices, these food items were categorized into the fruit group, and vegetable juices and tomato sauces were categorized into the vegetable group. Potatoes, pickled vegetables, and powdered vegetables were excluded because they are not considered a rich source of phytochemicals.

### Statistical analysis

Data analysis was conducted using the statistical package for social sciences (SPSS) software version 22 (IBM SPSS Statistics for Windows, Version 21.0. Armonk, NY: IBM Corp). The normality of the continuous variables was evaluated using the Kolmogorov‒Smirnov test. Normally and nonnormally distributed continuous variables are presented using the mean and standard deviation (SD), median, and interquartile range (IQR). Frequency and percentage were used to describe categorical variables. Student’s t test and Mann‒Whitney tests were used to compare normally and nonnormally distributed variables between groups. The chi-square test was used to compare continuous and categorical variables between study groups. Binary logistic regression assessed the relationship between study variables and the outcome variable (MS) by reporting the odds ratio (OR) and 95% confidence interval for OR [27]. The level of statistical significance was *p* < 0.05.

## Results

The study included 397 participants: 197 MS patients and 200 healthy controls. The data was complete for 174 (50.4%) MS patients and 171 (49.6%) healthy controls. The mean age was 32.45 years old. Table 1 presents the description and comparison of demographic and anthropometric characteristics between MS and healthy group. MS patients had a higher total body fat percentage (*p* = 0.031) and lower fat-free mass (*p* < 0.001) compared to healthy controls. The prevalence of smoking was significantly higher in the MS patient group (*p* = 0.01). There was no significant difference in gender distribution between the two groups.

**Table 1:** Comparison of demographic and anthropometric characteristics between MS patients and healthy controls

**Variable**	**MS Mean ± SD/Median (IQR)**	**Healthy Mean ± SD/Median (IQR)**	**p†**
Age (years)	32.00 (27.00–37.25)	31.00 (26.00–37.00)	0.296
BMI (kg/m^2^)	24.20 (21.10–27.43)	24.70 (21.60–29.00)	0.245
Total body fat (%)	28.80 ± 7.86	27.30 (19.90–33.90)	0.031*
Fat free mass (%)	40.80 (36.58–47.13)	45.30 (42.00–51.40)	< 0.001*
**Variable**	**MS n (%)**	**Healthy n (%)**	**p‡**
Smoking	40 (23.1%)	16 (9.4%)	0.001*

*SD* Standard Deviation, *IQR* Interquartile Range

^†^ The Mann‒Whitney test was used for the comparison

^‡^ The chi-square test was used for the comparison

^*^Significant difference

Description and comparison of the dietary intake of the MS patients and the healthy group are presented in Table 2. MS patients had significantly higher total energy intake compared to the healthy control group (*p* = 0.003). However, there were no significant differences between the two groups in the percentage of total energy intake from fiber, protein, carbohydrates, or fat.

**Table 2:** Comparison of dietary intake between MS patients and healthy controls

Variable	MS Mean ± SD/Median (IQR)	Healthy Mean ± SD/Median (IQR)	p
Energy (Kcal)	2508.25 (1843.91–3037.13)	2233.03 ± 837.29	0.003*†
Fiber (g)	19.49 (14.36–26.27)	17.40 (12.79–23.00)	0.056†
Protein (% of energy intake)	13.84 ± 2.57	13.87 ± 2.39	0.933‡
Fat (% of energy intake)	37.05 ± 6.18	37.42 (33.48–40.81)	0.842†
Carbohydrate (% of energy intake)	48.25 (45.03–53.21)	49.04 ± 6.33	0.989†

*SD* Standard Deviation, *IQR* Interquartile Range

^†^ The Mann‒Whitney test was used for the comparison

^‡^ Student’s t test was used for the comparison

^*^ Significant difference

Table 3 presents and compares the DPI, NEAP and PRAL scores between the MS patients and healthy group. The MS patient group had a significantly lower DPI score (*p* < 0.001) but significantly higher NEAP (*p* = 0.001) and PRAL (*p* < 0.001) scores compared to the healthy control group.

**Table 3:** Comparison of DPI, NEAP and PRAL scores between MS patients and the healthy group

Variable	MS Mean ± SD/Median (IQR)	Healthy Mean ± SD/Median (IQR)	p†
DPI	0.13 ± 0.07	0.16 (0.11–0.22)	< 0.001*
NEAP	51.36 ± 14.45	44.42 (38.01–53.06)	0.001*
PRAL	9.55 ± 14.28	2.96 (-4.82–12.02)	< 0.001*

*SD* standard deviation, *IQR* interquartile range, *DDS* dietary diversity score, *NEAP* net endogenous acid production, *PRAL* potential renal acid load

^†^ The Mann‒Whitney test was used for the comparison

^*^Significant difference

Relationship between MS and DPI, NEAP and PRAL scores are shown in Table 4. The study revealed a significant association between MS and sex, smoking, waist circumference, body fat percentage, fat-free mass percentage, energy intake, and DPI scores. Increased fat-free mass percentage and higher DPI scores were linked to 22.9% and 99.9% reductions in the chance of MS, respectively. Conversely, smoking, elevated waist circumference, higher body fat percentage, and greater energy intake were associated with 210.1%, 5.9%, 10.4%, and 14.3% increases in the chance of MS, respectively.

**Table 4:** Relationship between study variables and MS

variable	p	OR	95% CI for OR	
			**Lower**	**Upper**
**Age**	0.520	0.987	0.948	1.027
**Gender (Male)**	< 0.001*	0.018	0.004	0.078
**Smoking**	0.016*	3.101	1.0237	7.773
**BMI**	0.502	0.956	0.838	1.090
**Waist circumference**	0.044*	1.059	1.002	1.119
**Body fat (%)**	0.019*	1.104	1.016	1.199
**Fat free mass (%)**	< 0.001*	0.771	0.715	0.831
**Energy intake**	< 0.001*	1.143	1.072	1.220
**Fiber**	0.065	0.999	0.999	1.000
**Protein (% Cal)**	0.162	1.183	0.935	1.498
**Fat (% Cal)**	0.452	1.065	0.903	1.0257
**Carbohydrate (% Cal)**	0.220	1.119	0.935	1.341
**DPI**	< 0.001*	< 0.001	< 0.001	< 0.001
**NEAP**	0.974	1.001	0.959	1.044
**PRAL**	0.356	1.019	0.979	1.061

*DPI* Dietary phytochemical index, *NEAP* Net endogenous acid production, *PRAL* Potential renal acid load

^*^Significant relationship

## Discussion

Our study, involving 397 participants, provided a comprehensive analysis of the factors influencing the chance of MS. Notably, MS patients demonstrated a higher total body fat percentage and smoking prevalence, while the healthy group exhibited a higher fat-free mass. Despite similar macronutrient distributions across groups, MS patients had a significantly higher energy intake. Furthermore, our study found that MS patients had a lower DPI score but higher scores in the NEAP and PRAL. Our study also identified several factors that significantly influence the chance of developing MS, including sex, smoking habits, waist circumference, body fat percentage, fat-free mass percentage, and energy intake.

### Body composition and fat distribution

The findings reported in the current study align with previous investigations that have established a strong association between obesity and increased chance of MS. Several studies, as discussed in the review by Gianfrancesco and Barcellos [28], have consistently demonstrated a two-fold increased risk of developing MS in individuals with a BMI during adolescence and young adulthood. Specifically, the review highlighted findings from the Nurses' Health Study [29], where women with a BMI ≥ 30 kg/m2 at age 18 had a 2.25-fold increased risk of MS compared to those with a normal BMI. Similar observations were reported in population-based studies from Sweden [30], Norway, and Italy [31], further corroborating the link between elevated BMI and MS susceptibility.

In line with these previous reports, the current study found that individuals with a larger waist circumference had a 5.9% higher likelihood of developing MS, and the chance of MS increased by 10.4% with a one percent increase in body fat percentage. Conversely, a higher lean body mass percentage was associated with a reduced chance of MS 22.9% per one percent increase. These findings support the protective role of a lower body fat composition and higher lean mass against MS development, potentially attributed to the significantly higher energy intake observed in MS patients in the current study.

There are several links between adipose tissue and the immune system [32]. MS is characterized by inflammation and demyelination accompanied by axonal transection. An investigation indicates that a genetically elevated BMI is associated with an increased likelihood of developing MS, providing evidence for the causal involvement of obesity in the etiology of MS [33]. Furthermore, being overweight can worsen the severity of MS symptoms, make them harder to manage, increase the frequency of relapses, and speed up the progression of MS toward escalating disability.

### Gender differences in the chance of developing multiple sclerosis

Our study indicated gender as a significant factor that affected the chance of MS, and male sex was associated with a 90.2% reduction in the chance of developing MS. According to recent studies, MS is more prevalent in females than males [34]. In this regard, Greer and McCombe [34] demonstrated that MS is more prevalent in females than males. They suggest that the increased prevalence of MS in females could be attributed to several factors, including intrinsic differences between the male and female immune systems, genetic and epigenetic factors, effects of gonadal hormones, and environmental exposures.

### Smoking as a risk factor for multiple sclerosis

In line with Hedström’s study [35], our study revealed that smoking cigarettes increased the chance of MS by 210.1%. Cigarette smoking can cause oxidative stress and pro-inflammatory responses in lung tissue. Additionally, smoking can lead to posttranslational modifications of proteins in the lungs, which may affect their antigenicity and trigger autoimmunity against CNS antigens. So, the link between smoking and getting MS might be due to immune system responses against changed proteins that cross-react with antigens in the CNS [35].

### *Dietary phytochemical index and* chance of MS

In our study, we utilized DPI to measure dietary phytochemical content. We found that patients with MS had a lower DPI score than healthy participants. Our study showed that DPI was a protective factor that could be related to a reduced chance of MS. Phytochemicals and their derivatives can potentially protect the nervous system by regulating chronic inflammation, oxidative stress, and downstream signaling [15]. Studies have shown that phytochemicals can reduce mitochondrial dysfunction and inhibit the formation of α-synuclein accumulation-induced oxidative stress and inflammatory responses [16]. It is evident from various research studies that the Mediterranean Diet and Vegetarian Diet are dietary patterns characterized by a significant consumption of phytochemicals [36]. Several investigations have demonstrated that plant-based dietary patterns (whole grains, vegetables, legumes, nuts, and fruit) are highly adequate in augmenting the levels of phytochemicals in the bloodstream while concurrently diminishing the overall acid load of the diet [11, 12].

### *Dietary acid load and* chance of MS

Although the NEAP and PRAL scores were significantly higher among MS patients compared to the healthy group, there was no relationship between NEAP and PRAL and MS incidence. This could be due to changes in dietary intake among MS patients after their disease diagnosis, which may include increased consumption of fruits and vegetables, reduced amounts of saturated fat and sugar, and increased intake of dietary supplements. This result is contradictory to the study by Saeedirad et al. [37] in which higher DAL, as indicated by higher NEAP or PRAL scores, was associated with increased odds of MS.

According to several studies, a dietary pattern that increases the dietary acid load while low in phytochemicals leads to increased excretion of calcium and magnesium and cortisol secretion, ultimately resulting in decreased citrate excretion. These physiological changes are believed to contribute to elevated blood pressure and insulin resistance [19, 20]. Several articles highlight that insulin resistance and metabolic syndrome are more prevalent among MS patients [38], and this may be caused by the activation of microglia and elevated proinflammatory cytokines, which are known to be elevated in people with MS [18].

Additionally, consuming more phytochemical-rich plant foods such as fruits, vegetables, legumes, whole grains, nuts, seeds, and olive oil and reducing consumption of foods high in animal protein may help lower the risk of developing multiple sclerosis (MS). Other potential dietary and lifestyle recommendations that may help reduce the risk of MS include avoiding smoking, adopting a plant-based dietary pattern like the Mediterranean diet, and achieving a healthy body weight and body composition through balanced energy intake and physical activity levels. Optimizing the consumption of phytochemicals and acid–base balance through these dietary and lifestyle alterations may act as an additional preventive strategy in addition to traditional MS treatment options, while further study on dietary interventions is still needed.

There are limitations requiring mention. The study results could be impacted by recall bias, confounding variables, and the challenge of establishing causal relationships. Misclassification of dietary components may occur due to recall bias. At the same time, confounding factors such as genetic predisposition or environmental factors may obscure the underlying link between these items and chance of MS. Selection bias due to the specific locations the controls and cases were recruited from might limit the generalizability of the findings to other populations or geographic areas. Furthermore, the limited external validity, as the study was conducted on a specific Iranian population, might not apply to other ethnic or demographic groups due to differences in dietary habits, genetic backgrounds, and environmental exposures. Hence, it is imperative to interpret the study's findings in light of these potential biases and limitations. These limitations, however, underscore the need for further research in this area, building upon the foundation laid by this study.

## Conclusion

The present study revealed a higher prevalence of smoking and obesity, evidenced by an elevated total body fat percentage and a lower fat-free mass, among individuals with MS compared to their healthy counterparts. Concomitantly, MS patients exhibited a diminished DPI score, coupled with an increased DAL value. Optimizing nutritional strategies through the increased consumption of phytochemical-rich, plant-based foods and modulating the dietary acid–base balance may hold promise as an adjunctive therapeutic approach, complementing traditional treatment modalities for MS. The study provides valuable information on the potential links between diet and MS, but further research, particularly prospective cohort studies, are needed to confirm these findings and explore the mechanisms involved.

## Data Availability

The data supporting this study's findings are available from the corresponding author for all researchers interested in the subject matter upon reasonable request.

## Abbreviations

MS: Multiple sclerosis
CNS: Central nervous system
RRMS: Relapsing–remitting MS
FFQ: Food frequency questionnaire
BMI: Body mass index
WC: Waist circumference
DDS: Dietary Diversity Score
NEAP: Net Endogenous Acid Production
PRAL: Potential Renal Acid Load
DAL: Dietary Acid Load
